# Loss of BMP receptor type 1A in murine adipose tissue attenuates age-related onset of insulin resistance

**DOI:** 10.1007/s00125-016-3990-8

**Published:** 2016-05-21

**Authors:** Tim J. Schulz, Antonia Graja, Tian Lian Huang, Ruidan Xue, Ding An, Sophie Poehle-Kronawitter, Matthew D. Lynes, Alexander Tolkachov, Lindsay E. O’Sullivan, Michael F. Hirshman, Michael Schupp, Laurie J. Goodyear, Yuji Mishina, Yu-Hua Tseng

**Affiliations:** German Institute of Human Nutrition (DIfE), Department of Adipocyte Development and Nutrition, 114–116, Arthur-Scheunert Allee, 14558 Potsdam-Nuthetal, Germany; Section on Integrative Physiology and Metabolism, Joslin Diabetes Center, Harvard Medical School, One Joslin Place, Boston, MA 02215 USA; German Center for Diabetes Research (DZD), München-Neuherberg, Germany; Charité University School of Medicine, Institute of Pharmacology, Center for Cardiovascular Research, Berlin, Germany; Department of Biologic and Materials Sciences, School of Dentistry, University of Michigan, Ann Arbor, MI USA; Harvard Stem Cell Institute, Harvard University, Cambridge, MA USA

**Keywords:** Adipose tissue, Ageing, Bone morphogenetic proteins, Insulin sensitivity, Macrophage infiltration

## Abstract

**Aims/hypothesis:**

Adipose tissue dysfunction is a prime risk factor for the development of metabolic disease. Bone morphogenetic proteins (BMPs) have previously been implicated in adipocyte formation. Here, we investigate the role of BMP signalling in adipose tissue health and systemic glucose homeostasis.

**Methods:**

We employed the *Cre*/loxP system to generate mouse models with conditional ablation of BMP receptor 1A in differentiating and mature adipocytes, as well as tissue-resident myeloid cells. Metabolic variables were assessed by glucose and insulin tolerance testing, insulin-stimulated glucose uptake and gene expression analysis.

**Results:**

Conditional deletion of *Bmpr1a* using the *aP2* (also known as *Fabp4*)*-Cre* strain resulted in a complex phenotype. Knockout mice were clearly resistant to age-related impairment of insulin sensitivity during normal and high-fat-diet feeding and showed significantly improved insulin-stimulated glucose uptake in brown adipose tissue and skeletal muscle. Moreover, knockouts displayed significant reduction of variables of adipose tissue inflammation. Deletion of *Bmpr1a* in myeloid cells had no impact on insulin sensitivity, while ablation of *Bmpr1a* in mature adipocytes partially recapitulated the initial phenotype from *aP2-Cre* driven deletion. Co-cultivation of macrophages with pre-adipocytes lacking *Bmpr1a* markedly reduced expression of proinflammatory genes.

**Conclusions/interpretation:**

Our findings show that altered BMP signalling in adipose tissue affects the tissue’s metabolic properties and systemic insulin resistance by altering the pattern of immune cell infiltration. The phenotype is due to ablation of *Bmpr1a* specifically in pre-adipocytes and maturing adipocytes rather than an immune cell-autonomous effect. Mechanistically, we provide evidence for a BMP-mediated direct crosstalk between pre-adipocytes and macrophages.

**Electronic supplementary material:**

The online version of this article (doi:10.1007/s00125-016-3990-8) contains peer-reviewed but unedited supplementary material, which is available to authorised users.

## Introduction

Obesity is recognised as a significant risk factor for several of our most common medical conditions, such as type 2 diabetes mellitus and diseases associated with cardiovascular complications [1–3]. The majority of adipose tissue in the body is white adipose tissue (WAT), which stores energy as triacylglycerols and secretes adipokines [4]. The second type of fat, brown adipose tissue (BAT), expends energy in a process known as thermogenesis [5].

Normal adipose tissue displays a low-grade inflammation, which is presumably due to removal of apoptotic adipocytes. In obese individuals, WAT becomes a significant source of proinflammatory cytokines, which are known to promote systemic insulin resistance [6]. Specifically, increased infiltration of macrophages that surround the dead adipocytes, forming the so-called crown-like structures, is a source of these proinflammatory signals [7–9]. Recently, other immune cell populations, such as regulatory T cells and neutrophils, have also been implicated in these processes [10]. Adipose tissue-resident macrophages (ATMs) assume either proinflammatory or anti-inflammatory phenotypes termed M1 and M2, respectively. A general shift from a predominantly M2-like phenotype in healthy, lean WAT towards an M1 phenotype in inflamed, obese WAT is well documented [10, 11]. Generally, it should be noted that obesity leads to increased infiltration of all macrophage types, although accumulation of proinflammatory M1 ATMs greatly exceeds that of alternatively activated M2 ATMs [12, 13].

Bone morphogenetic proteins (BMPs) are members of the TGFβ protein superfamily. The role of BMPs in the regulation of adipose biology and energy metabolism has only recently become a field of interest [14–20]. Several BMPs are known to induce adipogenesis in a concentration-dependent manner; low concentrations promote adipogenesis while high concentrations are anti-adipogenic and, instead, promote osteochondrogenesis [21–24]. We recently discovered that BMP signalling plays an important role in the formation of brown adipocytes [15, 16, 25]. However, the role of BMPs in the physiological function of mature, adult WAT has not been addressed in detail. In our previous study, conditional deletion of the type 1A BMP receptor (*Bmpr1a*) using the *Myf5-Cre* driver led to a specific atrophy of interscapular BAT and compensatory browning of WATs, altogether establishing the metabolic equivalence of brite/beige adipose tissue and classical BAT [15]. To investigate BMP signalling in a broader spectrum of adipocytes, we deleted *Bmpr1a* in pre-adipocytes and adipocytes, targeting both BAT and WAT. Unexpectedly, the development of insulin resistance with increased age was prevented in knockout mice, suggesting that the role of BMP signalling in adipocyte function is highly context-dependent.

## Methods

A detailed description of the methods is included in the electronic supplementary material (ESM).

### Animals

All animal procedures were performed according to the Guide for the Care and Use of Laboratory Animals (http://grants.nih.gov/grants/olaw/Guide-for-the-Care-and-Use-of-Laboratory-Animals.pdf) and were approved by the Institutional Animal Care and Use Committee at Joslin Diabetes Center. Mice with *aP2-Cre-*driven deletion of the floxed *Bmpr1a* allele were generated and maintained as described previously [15, 26].

### Insulin tolerance testing

For the insulin tolerance test (ITT), mice were fasted for 2 h on the morning of the experiment before receiving an i.p. dose of 1.5 IU/(kg body weight) of recombinant human insulin (Humalog; Lilly, Indianapolis, IN, USA). Blood was collected from the tail vein for measurement of blood glucose levels before and 15, 30 and 60 min after injections.

### Glucose tolerance testing

Mice were fasted overnight (16 h) prior to i.p. injection of 2 g/(kg body weight) of glucose using a 20% (w/v) solution. Blood glucose was measured before and 15, 30, 60 and 120 min after injection.

### Serum analysis

Analyses of serum insulin, leptin, triacylglycerols, NEFA, TNFα and IL-6 were performed using standard colorimetric assays and ELISA procedures.

### Insulin-stimulated glucose uptake

The procedure was performed as described previously, with minor modifications (see ESM Methods) [27].

### Protein expression analysis

Analysis of gene expression on the protein level was performed as described previously [15]. Antibodies are specified in ESM Methods.

### Gene expression analysis

Total RNA isolation and gene expression analysis was conducted as described previously [15]. Primer sequences are listed in ESM Table 1.

### Analysis of adipocyte size

Adipocytes were analysed using ImageJ software (U.S. National Institutes of Health, Bethesda, MD) [28].

### Analysis of tissue-resident macrophages and blood monocytes

ATMs were analysed using FACS of freshly isolated stromal-vascular fractions of WAT as described previously [15].

### Analysis of physiology

Body composition, activity levels and energy expenditure were assessed as described previously [15].

### Cell culture

Pre-adipocytes were cultured as described previously [15]. Macrophages were collected from the peritoneal cavity of untreated, healthy mice.

### Statistical analysis

The data are presented as means ± SEM. Statistical significance was defined as *p* < 0.05 and determined by Student’s *t* test or two-way ANOVA when comparing multiple groups. In cases of unequal variance and non-normal distribution, non-parametric testing was conducted (Mann–Whitney *U* test).

## Results

### Loss of BMP receptor 1A in adipose tissue prevents age-related decline in insulin sensitivity

BMP signalling regulates early and late stages of adipocyte differentiation [20]. Therefore, we chose to use the *aP2* promoter to drive adipose-specific expression of Cre recombinase to generate a tissue-specific deletion of *Bmpr1a* in mouse adipose tissues (*aP2-Bmpr1a*-KO) [26]. As previously described, these mice displayed significantly reduced expression of *Bmpr1a* in BAT and WAT and a significant depletion of brown and brite/beige adipocytes [15]. Knockout mice were born smaller, had reduced bone length and maintained a trend of reduced body weight, lean mass and fat mass when body composition was analysed at 6 months of age on normal diet and after high-fat diet (HFD) feeding (ESM Fig. 1). Activity levels were not altered and energy expenditure tended to be reduced in *aP2-Bmpr1a*-KOs, but the latter was no longer apparent when normalised to body weight or lean mass (ESM Fig. 1). Histological evaluation of WAT revealed no changes in morphology, white adipocyte size or accumulation of fibrosis (ESM Fig. 2). Somewhat unexpectedly, we observed reduced expression and lower circulating levels of leptin, while expression of adiponectin remained unchanged (ESM Fig. 3). These findings suggest that lower leptin expression may be a direct effect of reduced BMP signalling rather than be due to reduced adipocyte size. In the absence of exogenous ligand treatment, we observed reduced phosphorylation of one of the main BMP target pathways, p38 mitogen-activated protein kinase (p38MAPK), but no changes of mothers against DPP homolog (SMAD)-1/5 phosphorylation in epididymal WAT (eWAT), whereas no changes in either pathway were observed in inguinal WAT (iWAT) (ESM Fig. 4).

**Fig. 1:**
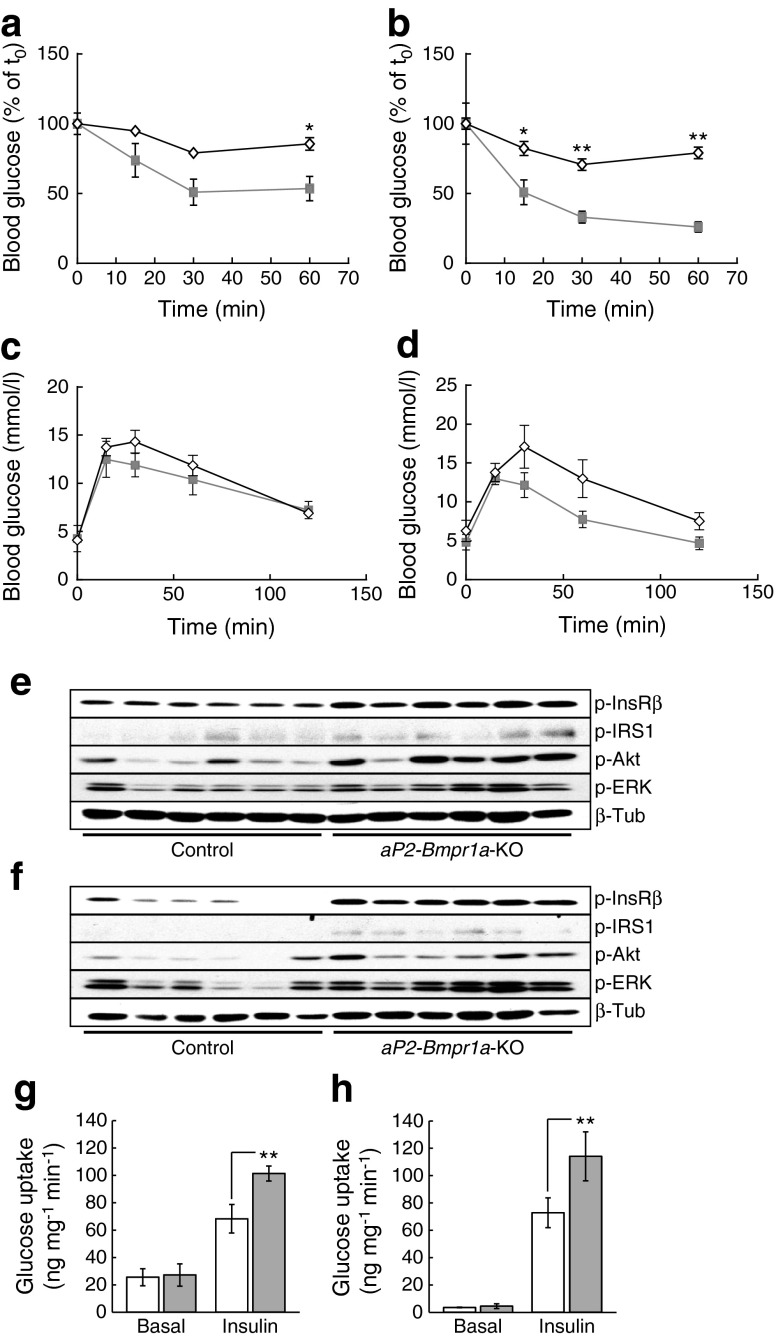
Loss of *Bmpr1a* in adipose tissue improves insulin sensitivity. (**a**, **b**) ITT in 38-week-old mice maintained on a normal chow diet (**a**) (AUC: *p* = 0.0286) or in 40-week-old mice maintained on 45%HFD from 4–5 weeks of age (**b**) (AUC: *p* = 0.0043). Diamonds, control mice; squares, *aP2-Bmpr1a-*KO mice. Data are shown as means ± SEM (*n* = 4 for both groups in **a**; *n* = 5 for control and *n* = 6 for knockout in **b**). *p < 0.05 and **p < 0.01 compared with control mice (**c**, **d**) GTT in 50-week-old mice fed either a normal chow diet (**c**) (AUC: *p* = 0.3429) or 45%HFD (**d**) (AUC: *p* = 0.6095). Diamonds, control mice; squares, *aP2-Bmpr1a-*KO mice. Data are shown as means ± SEM (*n* = 4 for both groups in **c**; *n* = 5 for control and *n* = 6 for knockout in **d**). (**e**, **f**) Western blot analysis of insulin-stimulated activation of the insulin signalling pathway in iWAT (**e**) and eWAT (**f**). Levels of the phosphorylated forms of insulin receptor-β (p-InsRβ), insulin receptor substrate (p-IRS)1, protein kinase B (p-Akt) and extracellular-signal regulated kinase (p-ERK) were detected and normalised to basal expression of β-tubulin (β-Tub). Quantification is shown in ESM Fig. 7. (**g**, **h**) Unstimulated (Basal) and insulin-stimulated glucose uptake (Insulin) in BAT (**g**) and tibialis anterior skeletal muscle (**h**). White bars, control mice; grey bars, *aP2-Bmpr1a-*KO mice. Data are shown as means ± SEM (*n* = 7 for basal control; *n* = 6 for basal knockout; *n* = 8 for insulin control; *n* = 6 for insulin knockout). **p < 0.01 compared with control mice

To analyse glucose homeostasis in more detail, we conducted ITTs and GTTs in mice either maintained on a normal diet or on an HFD containing 45% of energy from fat (45%HFD). Interestingly, *aP2-Bmpr1a*-KO mice on both diets displayed improved insulin sensitivity (Fig. 1a, b) and similar results were obtained for aged, but not young, mice maintained on 60%HFD (ESM Fig. 5). Glucose tolerance, on the other hand, showed a trend towards (but not significant) improvement on either diet when assessed at 52 weeks of age (Fig. 1c, d). Blood glucose, serum insulin and lipid levels remained unchanged at this age, although insulin levels tended to be lower in knockout mice on both diets (ESM Fig. 6).

To further explore this phenotype, we assessed the activation of the insulin signalling cascade following insulin stimulation. In this cohort, mice were maintained on 60%HFD until approximately 32 weeks of age. Consistent with the improved insulin sensitivity phenotype, phosphorylation of several members of the insulin signalling cascade was significantly enhanced in iWAT or eWAT of the knockout mice (Fig. 1e, f and ESM Fig. 7). BAT has recently been recognised as a significant glucose sink upon exposure to cold [29]. Despite the previously reported atrophy of BAT [15], *aP2-Bmpr1a*-KO mice displayed significant elevation of glucose uptake in the residual brown fat and skeletal muscle in response to insulin stimulation compared with their control littermates (Fig. 1g, h).

### Loss of *Bmpr1a* reduces proinflammatory gene expression and attenuates macrophage infiltration into adipose tissue

**Fig. 2:**
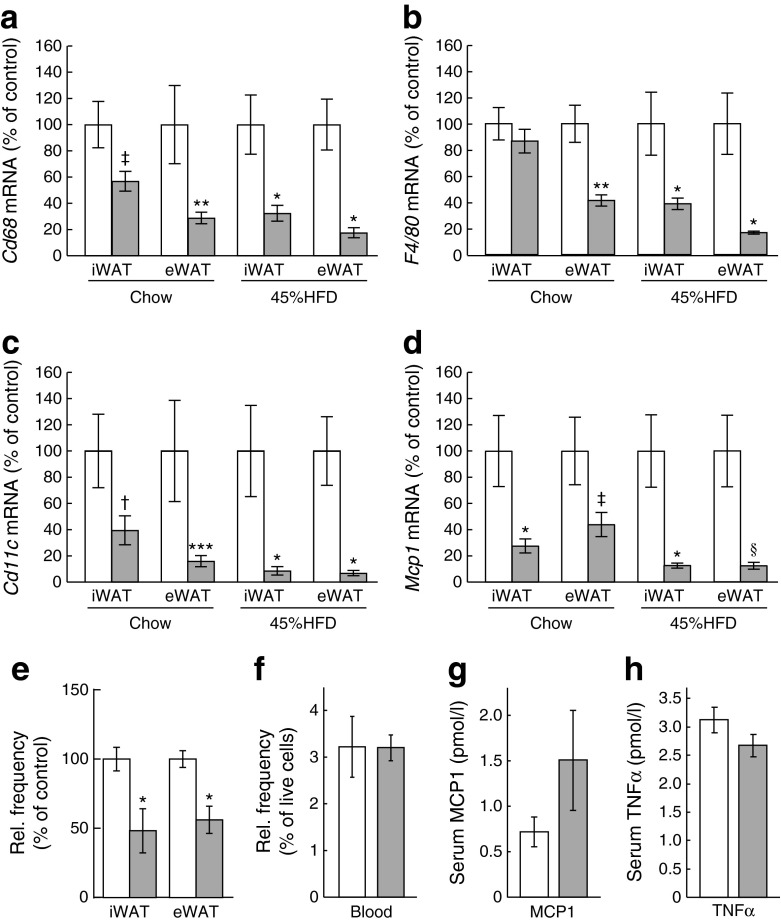
Loss of *Bmpr1a* protects adipose tissue from macrophage infiltration. (**a**–**d**) Gene expression analysis of macrophage markers *Cd68* (**a**), *F4/80* (**b**), *Cd11c* (**c**) and *Mcp1* (**d**) in iWAT and eWAT of mice maintained on chow diet or 45%HFD until 52 weeks of age. White bars, control mice; grey bars, *aP2-Bmpr1a-*KO mice. Data are shown as means ± SEM (*n* = 4–8 mice/group). (**e**, **f**) Flow-cytometric analysis of tissue-resident macrophages (surface markers: CD45^+^;CD11b^+^; F4/80^+^;CD3e^−^;CD19^−^;CD49b^−^;Ter119^−^) in iWAT and eWAT (**e**) (*n* = 6 for control and *n* = 5 for knockout) and circulating monocytes from whole blood (**f**) (*n* = 3 for both groups). (**g**, **h**) ELISA quantification of serum levels of MCP1 (**g**) and TNFα (**h**) (*n* = 6 for control; *n* = 4 for knockout). *p < 0.05, **p < 0.01, ***p < 0.001,^†^ *p* = 0.05, ^‡^ *p* = 0.065, ^§^ *p* = 0.067 compared with control mice of the same treatment group and/or tissue type

The link between insulin resistance and obesity-related adipose tissue immune cell infiltration is well established [30]. Therefore, we investigated whether expression of proinflammatory markers was reduced in *aP2-Bmpr1a*-KO mice. Indeed, gene expression of typical macrophage markers, such as *Cd68*, *F4/80* (also known as *Adgre1*), *Cd11c* (*Itgax*) and *Mcp1* (*Ccl2*), were significantly reduced in both inguinal and epididymal fat pads of *aP2-Bmpr1a*-KO mice maintained on either standard chow or a 45%HFD (Fig. 2a–d). Similar trends were also observed in mice maintained on a 60%HFD until 1 year of age, while no differences in inflammatory gene expression were observed in young mice on normal diet (ESM Fig. 8). To address the role of macrophages in this phenotype, we next quantified macrophage infiltration. Infiltration with CD45^+^/CD11b^+^/F4/80^+^ macrophages in WAT of knockout mice was significantly diminished whereas frequencies of peripheral blood monocytes were unchanged, suggesting that reduced macrophage infiltration occurred within the adipose tissue (Fig. 2e, f). This was consistent with the unchanged levels of the circulating proinflammatory cytokines monocyte chemotactic protein 1 (MCP1) and TNFα (Fig. 2g, h).

**Fig. 3:**
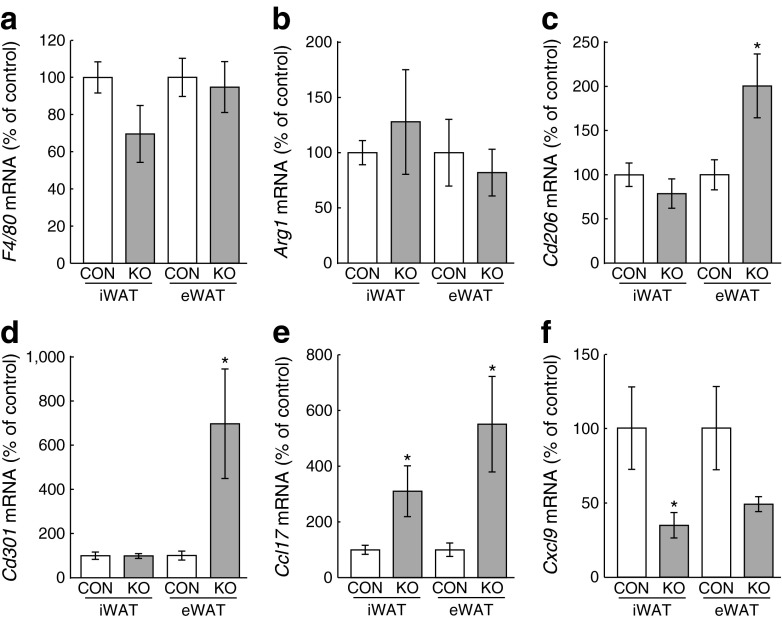
Anti-inflammatory polarisation of ATMs in *Bmpr1a*-deficient adipose tissue. Gene expression analysis of *F4/80* (**a**), *Arg1* (**b**), *Cd206* (**c**), *Cd301* (**d**), *Ccl17* (**e**) and *Cxcl9* (**f**) in FACS-purified macrophages (surface markers: CD45^+^;CD11b^+^;F4/80^+^;CD3e^−^;CD19^−^;CD49b^−^;Ter119^−^) isolated from mouse iWAT and eWAT. White bars, control mouse macrophages; grey bars, *aP2-Bmpr1a-*KO mouse macrophages. Data are shown as means ± SEM. Macrophage isolation experiments were repeated with two or three mice/genotype and two or three independent experiments were carried out for mice maintained on an HFD. mRNA yield from sorted macrophages was limited and gene expression data from the individual experiments were pooled for statistical analysis (*n* = 9 for control and *n* = 8 for knockout in **a**, **e**, and **f**; control: *n* = 6 for control and *n* = 5 for knockout in **b**–**d**). *p < 0.05 compared with control mice of the same tissue type

To determine whether macrophage activation was altered in *aP2-Bmpr1a*-KO mice, we next quantified expression of typical macrophage activation markers [31, 32]. To this end, we isolated macrophages from both WAT depots by flow cytometry. While mRNA levels of a general macrophage marker (*F4/80*) and a well-established M2 marker (*Arg1*) were unchanged (Fig. 3a, b), expression of other M2 markers (*Cd206* [*Mrc1*] and *Cd301* [*Clec10a*]) was upregulated in eWAT, but not iWAT, of *aP2-Bmpr1a*-KO mice (Fig. 3c, d). Accordingly, expression levels of the M2-related *Ccl17* and the M1-related *Cxcl9* were upregulated and downregulated, respectively (Fig. 3e, f) [32]. Expression of other established M1 markers, such as *Il1b* or inducible nitric oxide synthase, were not altered (data not shown).

### Loss of BMP signalling in myeloid cells does not affect insulin sensitivity

**Fig. 4:**
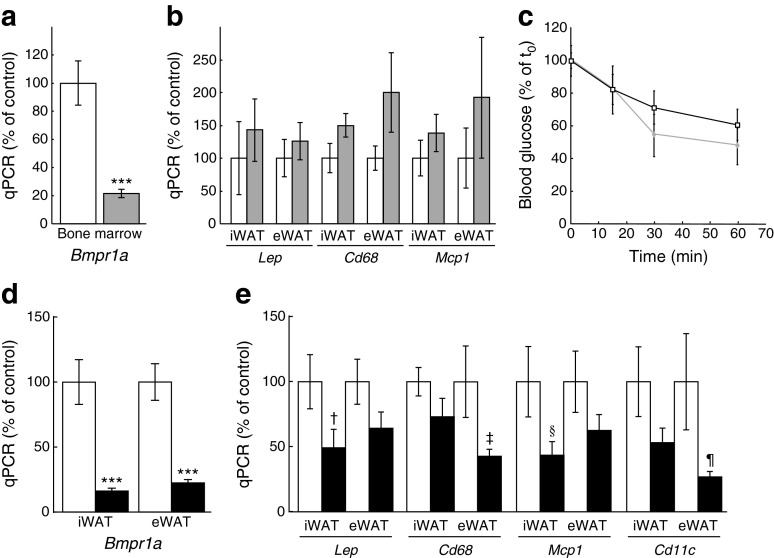
Loss of *Bmpr1a* in adipocytes, but not myeloid cells, reduces macrophage infiltration. (**a**) Gene expression analysis of *Bmpr1a* mRNA in bone marrow of mice with *LysM-Cre-*driven deletion of *Bmpr1a* (*LysM-Bmpr1a*-KO). White bars, control mice; grey bars, *LysM-Bmpr1a*-knockout mice. Data are shown as means ± SEM (*n* = 3/group). (**b**) mRNA levels of leptin and macrophage infiltration markers *Cd68* and *Mcp1* in WAT depots of *LysM-Bmpr1a*-KO mice. White bars, control mice; grey bars, *LysM-Bmpr1a*-knockout mice. Data are shown as means ± SEM (*n* = 3/group). (**c**) ITT in HFD-fed *LysM-Bmpr1a*-KO mice at 52 weeks of age. Squares, control mice; diamonds, *LysM-Bmpr1a*-KO mice. Data are shown as means ± SEM (*n* = 7 mice/group). (**d**) *Bmpr1a* mRNA levels in WAT depots of knockout mice with *Adipoq-Cre-*driven deletion of *Bmpr1a* (*Adipoq-Bmpr1a*-KO). White bars, control mice; black bars, *Adipoq-Bmpr1a*-KO mice. Data are shown as means ± SEM (*n* = 7 for control and *n* = 6 for knockout). (**e**) mRNA levels of leptin and macrophage infiltration markers *Cd68*, *Mcp1* and *Cd11c* in WAT of *Adipoq-Bmpr1a*-KO mice. Data are shown as means ± SEM (*n* = 7 for control and *n* = 6 for knockout). ***p < 0.001, ^†^ *p* = 0.078, ^‡^ *p* = 0.084, ^§^ *p* = 0.096, ^¶^ *p* = 0.097 compared with control mice of the same treatment group and/or tissue type. qPCR, quantitative real-time PCR

Previous studies have demonstrated that *aP2* is also expressed in cell types other than adipocytes. Specifically, it is also expressed in macrophages, where aP2 plays a role in foam cell formation [33]. These findings raise the possibility that use of *aP2-Cre* may also result in gene deletion in macrophages infiltrating the adipose tissue. While this possibility is still valid, a recent study using the same *aP2-Cre* strain as us showed no Cre-mediated recombination in adipose tissue macrophages [34]. Consistent with this report, *Bmpr1a* mRNA levels were not changed in macrophages sorted from WAT of *aP2-Bmpr1a*-KO mice when compared with WAT from control mice (ESM Fig. 9). Nevertheless, to determine whether loss of BMP signalling in macrophages could still be responsible for reduced adipose tissue macrophage infiltration and improved insulin sensitivity, we generated a mouse model with myeloid-specific ablation of BMP receptor 1A (BMPR1A) using the *LysM* (also known as *Lyz2*)*-Cre* mouse strain [35]. Efficient ablation of BMPR1A expression was observed in tissues with a high content of myeloid cells, such as bone marrow, in *LysM-Bmpr1a*-KO mice (Fig. 4a). In this strain, body and tissue weights were unchanged (data not shown) and gene expression levels of *Lep*, *Cd68* and *Mcp1*, which were significantly decreased in the *aP2-Bmpr1a*-KO mice, were unchanged in iWAT and eWAT of 5-month-old mice (Fig. 4b). Moreover, insulin sensitivity was not altered in 12-month-old *LysM-Bmpr1a*-KO mice compared with control mice under high-fat feeding (Fig. 4c). Hence, the improved insulin sensitivity in *aP2-Bmpr1a*-KO mice cannot be attributed to deletion of *Bmpr1a* in macrophages.

### Loss of *Bmpr1a* in mature adipocytes improves the inflammatory gene expression profile

To determine whether the improved insulin sensitivity in *aP2-Bmpr1a*-KO mice can be directly linked to adipocyte-specific changes, we generated a third mouse model using the *Adipoq*-driven *Cre* mouse strain (*Adipoq-Cre*). Unlike *aP2-Cre*, which also causes recombination in adipogenic progenitor cells [36], *Adipoq-Cre* is expressed exclusively in mature adipocytes, thus targeting a more restricted population of cells within WAT [34]. In *Adipoq-Bmpr1a*-KO mice, decreased *Bmpr1a* mRNA levels were observed in WAT (Fig. 4d). Further analysis revealed a trend towards decreased gene expression of *Lep* and macrophage markers *Cd68*, *Mcp1* and *Cd11c* (Fig. 4e). However, body weight, adipose tissue weight and insulin sensitivity analysed by GTT or ITT remained unaltered in *Adipoq-Bmpr1a*-KO mice on a normal diet or after 5 months of high-fat feeding (ESM Fig. 10).

### Loss of *Bmpr1a* in pre-adipocytes directly affects activation and cytokine expression patterns in macrophages

**Fig. 5:**
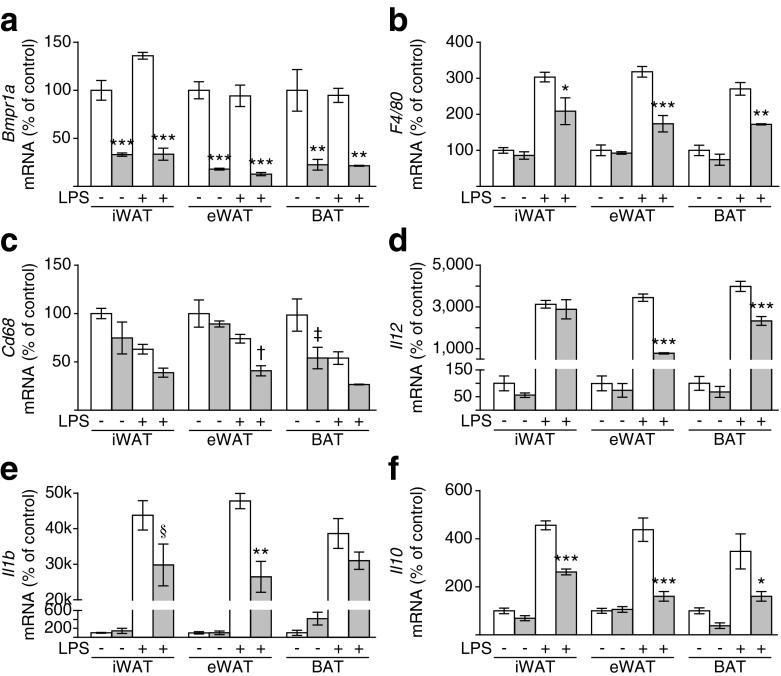
Co-cultivation of macrophages with *Bmpr1a*-KO pre-adipocytes reduces macrophage activation. Gene expression analysis of *Bmpr1a* (**a**), *F4/80* (**b**), *Cd68* (**c**), *Il12* (**d**), *Il1b* (**e**) and *Il10* (**f**) was carried out in co-cultures of macrophages with pre-adipocytes isolated by flow cytometry from iWAT, eWAT and BAT of *Bmpr1a*-KO mice. White bars, pre-adipocytes infected with adenovirus expressing the gene for green fluorescent protein (control); grey bars, pre-adipocytes infected with adenovirus expressing *Cre* to cause deletion of the floxed *Bmpr1a* allele. Macrophages were isolated from non-floxed wild-type mice and co-cultured with pre-adipocytes for 24 h alone (−) or in the presence of LPS (+). Data are shown as means ± SEM (*n* = 3).*p < 0.05, **p < 0.01, ***p < 0.001, ^†^ *p* = 0.07, ^‡^ *p* = 0.06, ^§^ *p* = 0.09 compared with control cells of the same treatment group as assessed by ANOVA for each tissue type separately

To determine whether interactions between macrophages and adipocyte progenitors could be responsible for the more pronounced phenotype of the *aP2-Bmpr1a*-KO mice, we used a co-culture approach (see ESM Fig. 11a for experimental scheme). Macrophages were isolated from wild-type C57BL/6J mice and pre-adipocytes were isolated from mice carrying a homozygous floxed *Bmpr1a* allele. Isolated pre-adipocytes were infected with adenoviruses either expressing green fluorescent protein or *Cre* recombinase to generate pre-adipocytes with intact or impaired BMP signalling, respectively. Lipopolysaccharide (LPS) was added to the co-culture to activate expression of inflammatory cytokines from macrophages (ESM Fig. 11). Four days post infection, control or *Bmpr1a*-deficient progenitor cells isolated from white and brown adipose depots were co-cultured with macrophages for 24 h. Measurement of *Bmpr1a* mRNA levels showed a significant reduction in co-cultures with *Cre*-infected pre-adipocytes and pure cultures of *Cre*-infected pre-adipocytes (Fig. 5a and ESM Fig. 11b). Importantly, expression of macrophage-specific marker genes (such as *F4/80* and *Cd68*) and LPS-induced cytokines (*Il1b*, *Il10* and *Il12*) was significantly reduced in co-cultures of knockout pre-adipocytes and macrophages (Fig. 5b–f). These data provide a potential cellular mechanism for the phenotypes observed in *aP2-Bmpr1a*-KO mice.

## Discussion

In the present study, we address the physiological effects of adipose tissue BMP signalling on glucose homeostasis and insulin sensitivity. We report that WAT displays a marked reduction in macrophage infiltration and improved insulin sensitivity, which develops with increased age in mice with adipose-specific deletion of *Bmpr1a*. In those mice, the response to insulin stimulation is enhanced locally within the adipose tissue, as well as at the systemic level, as signified by improved insulin sensitivity and elevated insulin-stimulated glucose uptake in skeletal muscle. This phenotype can be explained, at least in part, by reduced macrophage infiltration into WAT and reduced proinflammatory polarisation of ATMs due to loss of BMP signalling in the adipocytic lineage. Importantly, myeloid-specific deletion of *Bmpr1a* does not affect WAT inflammation or insulin sensitivity, indicating that reduced BMP signalling in macrophages does not contribute to this phenotype.

We previously demonstrated that loss of BMPR1A specifically impairs brown adipogenesis [15]. This occurred in classical interscapular BAT using a *Myf5-Cre* driver and, similarly, in *aP2-Bmpr1a*-KO mice where an impaired formation of brown and brite/beige adipocytes was observed [15]. The novel findings presented here are surprising since it is commonly assumed that brown adipocytes confer beneficial metabolic features and promote an insulin-sensitive state. For instance, transplantation of BAT to the visceral cavity of mice resulted in a marked improvement of glucose tolerance [37]. Consistent with these data, the residual BAT in *aP2-Bmpr1a*-KO mice appears to be more insulin sensitive and this could offset the overall effects of BAT atrophy to some degree. On the other hand, it is well known that immune cells and proinflammatory processes play a major role in the development of insulin resistance [38]. Interestingly, increased expression of *Bmpr1a* in WAT correlates with insulin resistance in human obesity, as reported by Boettcher et al [39]. This study also reports that individuals with impaired glucose tolerance or overt diabetes show increased expression levels of *BMPR1A* in WAT [39]. These findings support the notion that changed expression levels of *BMPR1A* in WAT could regulate insulin sensitivity.

Since *aP2-Cre* potentially deletes *Bmpr1a* in macrophages in addition to adipogenic cells, an important aspect of our study is to determine whether loss of *Bmpr1a* in either the adipogenic or myeloid lineages leads to improved insulin sensitivity. However, consistent with a previous report [34], we found expression of *Bmpr1a* in macrophages isolated from WAT of *aP2-Bmpr1a*-KO mice to be unchanged. Additionally, deletion of *Bmpr1a* in myeloid cells, which include macrophages, does not recapitulate the phenotype of the *aP2*-driven knockouts. The role of BMPs in inflammatory processes is rather complex. Some studies have reported anti-inflammatory effects on macrophages and other immune cells [40, 41], while others show that active BMP signalling may promote inflammation, a process that seems to be highly ligand-specific [42].

In a previous study it was reported that *aP2-Cre* is active in the heart and interstitial cells of the skeletal muscle [34]. Muscle-resident interstitial cells are known to possess high adipogenic potential and are involved in myogenic regeneration [25, 43]. This could therefore help explain the improved insulin sensitivity observed in muscle of *aP2-Bmpr1a*-KO mice. Alternatively, an endocrine effect of a healthier adipose tissue releasing different adipokines that affect muscle insulin sensitivity is possible. As the *Adipoq-Bmpr1a*-KO strain only partially recapitulates the phenotype of the *aP2-Cre* driven knockout mice, these findings, taken together, suggest that reduced BMP signalling in adipogenic progenitor cells is a key factor in this process. This supposition is strongly supported by our observation that co-cultivation with *Bmpr1a*-deficient pre-adipocyte blunts expression of proinflammatory markers. It is thus conceivable that the phenotype observed in *aP2-Bmpr1a*-KO mice is due to loss of BMP signalling within the adipogenic lineage, comprised of pre-adipocytes and mature adipocytes. It is also conceivable that the differences in manifestation of the phenotype using the two adipose-specific *Cre*-lines are related to differences in the timing of *Cre* expression in both models that occurs later (i.e. only in mature adipocytes in the *Adipoq-Cre* strain). Thus, alterations of signalling through BMPR1A during the earlier stages of white adipocyte differentiation, rather than in fully mature adipocytes, could be critical to the reduction of proinflammatory signals and improved insulin sensitivity.

‘Inflamm-ageing’ is a concept encompassing age-related deterioration of the innate immune system response, low-grade chronic inflammation and the onset of age-related pathologies such as insulin resistance [44]. In a well-described vicious circle, chemoattractants originating from senescent adipocytes and pre-adipocytes promote increased infiltration by proinflammatory immune cells, which in turn exacerbate the negative metabolic properties of adipocytes [45]. Thus, altered BMP signalling in adipogenic cells might affect the release of adipokines that regulate recruitment to and function of immune cells within adipose tissue. In aged animals, predominantly proinflammatory immune cells (i.e. M1 macrophages) are recruited and reducing infiltration with these immune cells would, therefore, attenuate the development of insulin resistance [46]. In addition, ageing is accompanied by a switch from M2 anti-inflammatory macrophages towards proinflammatory M1 macrophages [47]. Hence, a model such as the *aP2-Bmpr1a*-KO mouse, where overall macrophage infiltration into adipose tissue is reduced, could retain a healthier metabolic profile due to a general lack of infiltrating immune cells.

In summary, our study provides new insight into the role of BMP signalling in maturing white adipocytes. In brown adipocytes, BMPs are critical for formation and thermogenic activity, whereas in white adipocytes, BMP signalling appears to regulate the endocrine interaction between cells of the adipose lineage and immune cells. A better understanding of these processes could help decipher the intricate crosstalk between adipocytes and other adipose tissue-resident cell types and this could provide novel avenues to counter the progression and pathology of insulin resistance.

## Electronic supplementary material

Below is the link to the electronic supplementary material.

ESM (PDF 584 kb)

## Abbreviations

ATM: Adipose tissue-resident macrophage
BAT: Brown adipose tissue
BMP: Bone morphogenetic protein
BMPR1A: BMP receptor 1A
eWAT: Epididymal WAT
HFD: High-fat diet
ITT: Insulin tolerance test
iWAT: Inguinal WAT
LPS: Lipopolysaccharide
MCP1: Monocyte chemotactic protein 1
WAT: White adipose tissue
