# Supplementary material for: Loss of BMP receptor type 1A in murine adipose tissue attenuates age-related onset of insulin resistance
Source: Diabetologia. 2016 May 21;59:1769–77. doi: 10.1007/s00125-016-3990-8 (PMC4930470; doi:10.1007/s00125-016-3990-8)
Supplement: Supplementary file 1 — (PDF 584 kb) [file 125_2016_3990_MOESM1_ESM.pdf]

Electronic supplementary material (ESM): Methods

Animals. Mice with aP2-Cre-driven (strain: Tg(Fabp4-cre)#Abel) deletion of the floxed *Bmpr1a*-allele (strain: Bmpr1atm2.1Bhr) was generated and maintained as described before [15, 26]. Other Cre-recombinase expressing mouse lines were obtained from The Jackson Laboratory (Bar Harbor, ME): *Adipoq-Cre* (strain: B6;FVB-Tg(Adipoq-cre)1Evdr/J; stock 010803) and *LysM-Cre* (strain: B6.129P2-Lyz2tm1(cre)Ifo/J; stock 004781).

Insulin stimulated glucose uptake. Mice were fasted for approximately 16 h overnight (5 pm – 9 am) and anesthetized with pentobarbital sodium (90 mg/kg body weight, i.p.). After 30 min, baseline blood samples were collected from the tail vein. An intravenous bolus of 2-deoxy-D-[^{3}H]-glucose (33 µCi/kg lean body weight) was administered alone in saline or, for maximal insulin stimulation, in combination with insulin (16.6 U/kg lean body weight.) through the retro-orbital sinus together with a 20% w/v glucose bolus (1.25 g/kg lean body weight) to prevent hypoglycaemia, for basal and insulin stimulated groups. Basal- and insulin-stimulated animals were killed 45 and 15 min after tracer injection, respectively. Tissues were removed and snap-frozen in liquid nitrogen. Glucose uptake was corrected for time after tracer injection. White adipose tissues were used for western blot analysis of insulin signalling activation whereas brown adipose tissue, skeletal muscle, and other tissues were used for analysis of glucose uptake. Accumulation of [^{3}H]2-deoxyglucose-6-P was assessed in tissues using a perchloric acid/BaOH-ZnSO4 precipitation procedure described previously.

Protein expression analysis. To detect individual proteins, antibodies were used as follows: Anti-phospho-insulin receptor-beta (p-InsRβ), anti-phospho-protein kinase B (p-Akt), anti-phospho-extracellular-regulated kinase (p-ERK), anti-beta tubulin (β-Tub), anti-phospho-Smad (p-Smad-)1/5, anti-Smad1, anti-phospho-p38 mitogen activated protein kinase (p-p38), anti-p38 mitogen activated protein kinase (p38; all from Cell Signaling Technologies, Danvers, MA or Cell Signaling Technologies/New England Biolabs GmbH, Frankfurt am Main, Germany; all used in 1:1000 dilutions), anti-phospho-insulin receptor substrate-1 (p-IRS1; from Life Technologies,

Carlsbad, CA), and anti-beta-actin (β-Actin; Sigma-Aldrich, Saint Louis, MO or Munich, Germany).

Analysis of adipocyte size. Adipocytes were analysed using ImageJ software (29). In brief, images were collected at 200x magnification using the same microscope setting for all captions for related batches of tissue sections. Image channels were split and the green channel was used for subsequent analysis. Images were inverted and background was subtracted (rolling ball radius of 30.0 pixels). In subsequent steps, the image was adjusted so that membranes became binary black borders surrounding adipocytes that were quantified using the automatic area recognition tool of the program. Broken adipocytes that would connect to areas including more than one adipocyte were deselected manually. Per tissue sample, two to three independent sections were prepared. Two or three captions from different areas were collected from each section for quantification and used to calculate average adipocyte area sizes per animal for subsequent statistical analyses.

Analysis of tissue-resident macrophages and blood monocytes. ATMs were analysed using FACS. Freshly isolated stromal-vascular fractions (SVF) of WAT were subjected to antibody staining and FACS-selection as follows: To identify macrophages, positive selection for anti-CD45 (1:200), anti-F4/80 (1:50), and anti-CD11b (1:200) was employed, as well as negative selection for anti-CD3e (1:100; T cells), anti-CD19 or anti-B220 (1:200 or 1:100, respectively; B cells), anti-Ter119 (1:100; erythrocytes); and anti-CD49b (1:100; pan-NK cells) (all antibodies from eBioscience-Affymetrix, San Diego, CA or Frankfurt am Main, Germany). For determination of blood monocytes levels, approximately 100 µl of blood were collected from the tail vein into 10 ml of ice-cold PBS containing 5 µmol/l of EDTA. Erythrocytes were lysed and antibody staining and FACS-analysis as above.

Cell culture and preadipocytes/macrophage co-culture. Pre-adipocytes were isolated from animals carrying homozygous floxed Bmpr1a-alleles using flow cytometry as described previously using positive selection for expression of surface marker stem cell antigen (Sca)-1,

and negative selection for the surface proteins CD45 and CD31 (PECAM) [15]. All cells were cultured in DMEM/F12 supplemented with 10% fetal bovine serum. Macrophages were collected by peritoneal lavage of untreated wildtype C57BJ/6-L mice two days after sorting of the pre-adipocytes. To this end, animals were killed by cervical dislocation. A small incision was created in the abdominal skin of which was then removed by pulling the incision apart. Great was taken not to injure the peritoneum. A volume of approximately 5 ml PBS was injected into the peritoneal cavity without causing injuries to the inner organs. Animals were agitated gently to move the fluid within the cavity. Subsequently, a blunt-ended syringe tip was used to re-aspirate the buffer. Aspirates from several animals were combined and washed by centrifugation and cultured in the presence of 50 ng/ml macrophage colony-stimulating factor (M-CSF, from PeproTech, Hamburg, Germany) and 50 µg/ml gentamicin (from Sigma-Aldrich, Munich, Germany). On the same day, pre-adipocytes were infected with adenovirus at approximately 4,00E+07/ml infectious units to express either GFP or Cre-recombinase. After overnight infections, virus-containing supernatants were removed and cells were washed once and allowed to recover for four days. For co-cultures, 30,000 pre-adipocytes were seeded together with 15,000 macrophages into 48-well cell culture plates and were either left untreated or supplemented with 10 ng/ml lipopolysaccharide (LPS) for 24 h prior to harvest. Cells were harvested, RNA was extracted and gene expression was assessed using standard protocols as described before [15].

ESM Table 1 (Gene expression primers)

**Target gene**	**Forward primer**	**Reverse primer**
Adipoq	GGCAGGAAAGGAGAACCTGG	AGCCTTGTCCCTCTTGAAGAG
Arg1	AGACCACAGTCTGGCAGTTG	CCACCCAAATGACACATAGG
Bmpr1a (Alk3)	CAGCAGGACCAGTCATTCAA	CTGGCTTCTTCTGGTCCAAG
Ccl17	AGGGATGCCATCGTGTTTCT	CCTTGGGTTTTTCACCAATCT
Cd11c	CAGAACTTCCCAACTGCACA	TCTCTGAAGCTGGCTCATCA
Cd206	TGATTACGAGCAGTGGAAGC	GTTCACCGTAAGCCCAATTT
Cd301	GGAAGCCAAGACTTCACACA	CTCTTCCCGCTCCAAGTTCT
Cd68	GCAGCACAGTGGACATTCAT	TTGCATTTCCACAGCAGAAG
Cxcl9	CGATCCACTACAAATCCCTCA	TAGGCAGGTTTGATCTCCGT
F4/80 (Emr1)	TTTCCTCGCCTGCTTCTTC	CCCCGTCTCTGTATTCAACC
Il1b	AGTTGACGGACCCCAAAAG	AGCTGGATGCTCTCATCAGG
Il10	CAGAGCCACATGCTCCTAGA	TGTCCAGCTGGTCCTTTGTT
Il12	CTAGACAAGGGCATGCTGGT	GCTTCTCCCACAGGAGGTTT
Lep	CCTCATCAAGACCATTGTCACC	TCTCCAGGTCATTGGCTATCTG
Mcp1 (Ccl2)	AGGTCCCTGTCATGCTTCTG	TCATTGGGATCATCTTGCTG

ESM Fig. 1

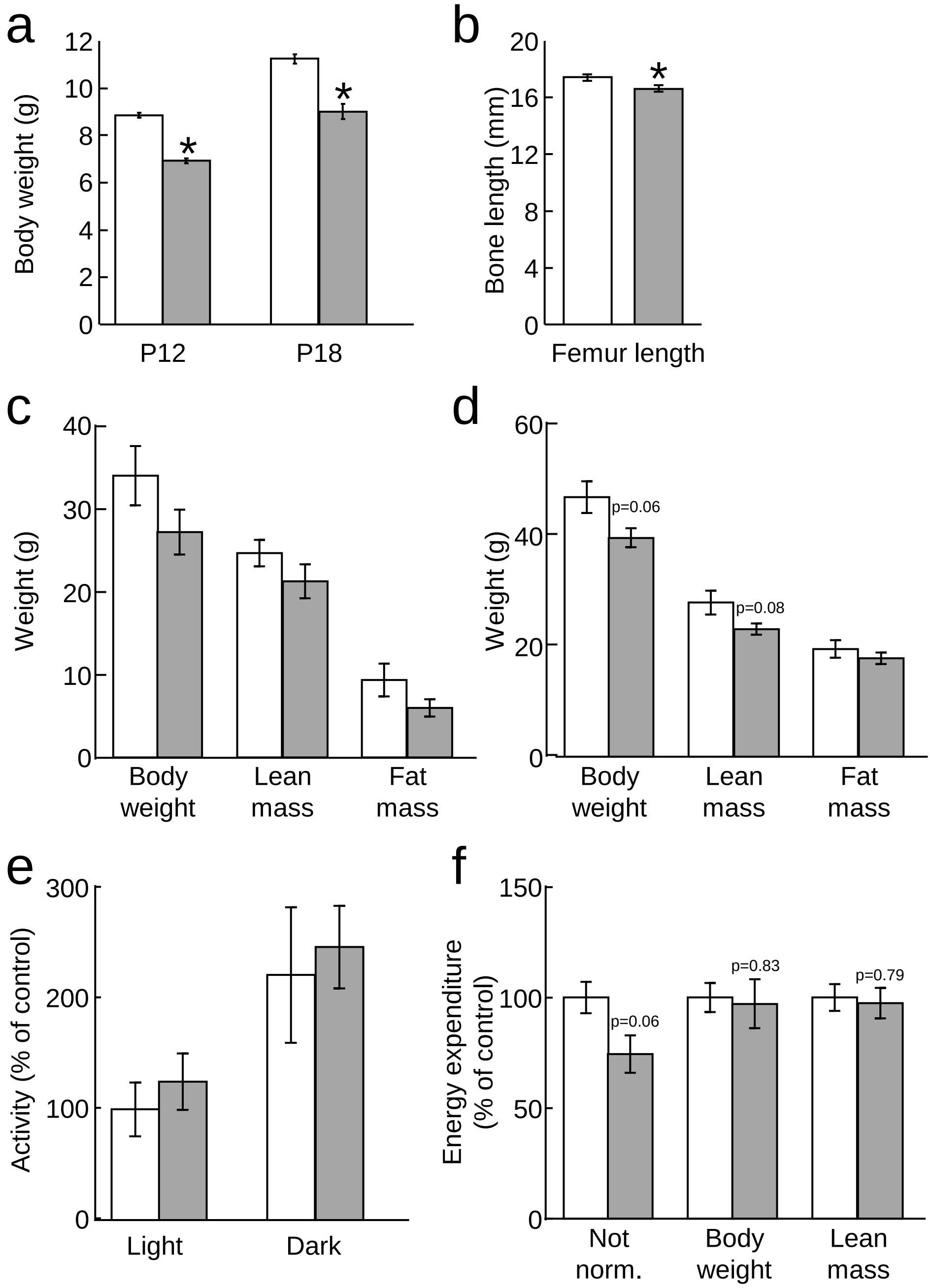
a
b
c
d
e
f
Body weight (g)
P12
P18
Bone length (mm)
Femur length
Weight (g)
Body weight
Lean mass
Fat mass
p=0.06
p=0.08
Activity (% of control)
Light
Dark
Energy expenditure (% of control)
Not norm.
p=0.83
p=0.79

ESM Fig. 1

ESM Figure 1 *aP2-Bmpr1a*-KO display reduced body size but unchanged activity and energy metabolism. (**a**) Body weights 12 (P12) and 18 (P18) days after birth. White bars represent controls, grey bars represent *aP2-Bmpr1a*-KO mice (n=5-6 for control and n=4 for knockout). Data are shown as means ± SEM (applies to all subsequent panels). (**b**) Femur length in 8-weeks-old control and *aP2-Bmpr1a*-KO mice (n=8/group). Body composition to determine lean and fat masses was assessed by dual-energy X-ray absorptiometry (DEXA) in mice maintained on a normal diet (**c**) (n=6 for control and n=4 for knockout) and on an 60%HFD (**d**) (n=5/group) at 6 and 4 months of age, respectively. Activity levels during light and dark phases (**e**) and energy expenditure (**f**) in mice maintained on a normal diet without normalisation or after normalisation to body weight or lean mass (n=4/group). *p<0.05 compared with control mice of the same treatment group and/or tissue type.

ESM Fig. 2

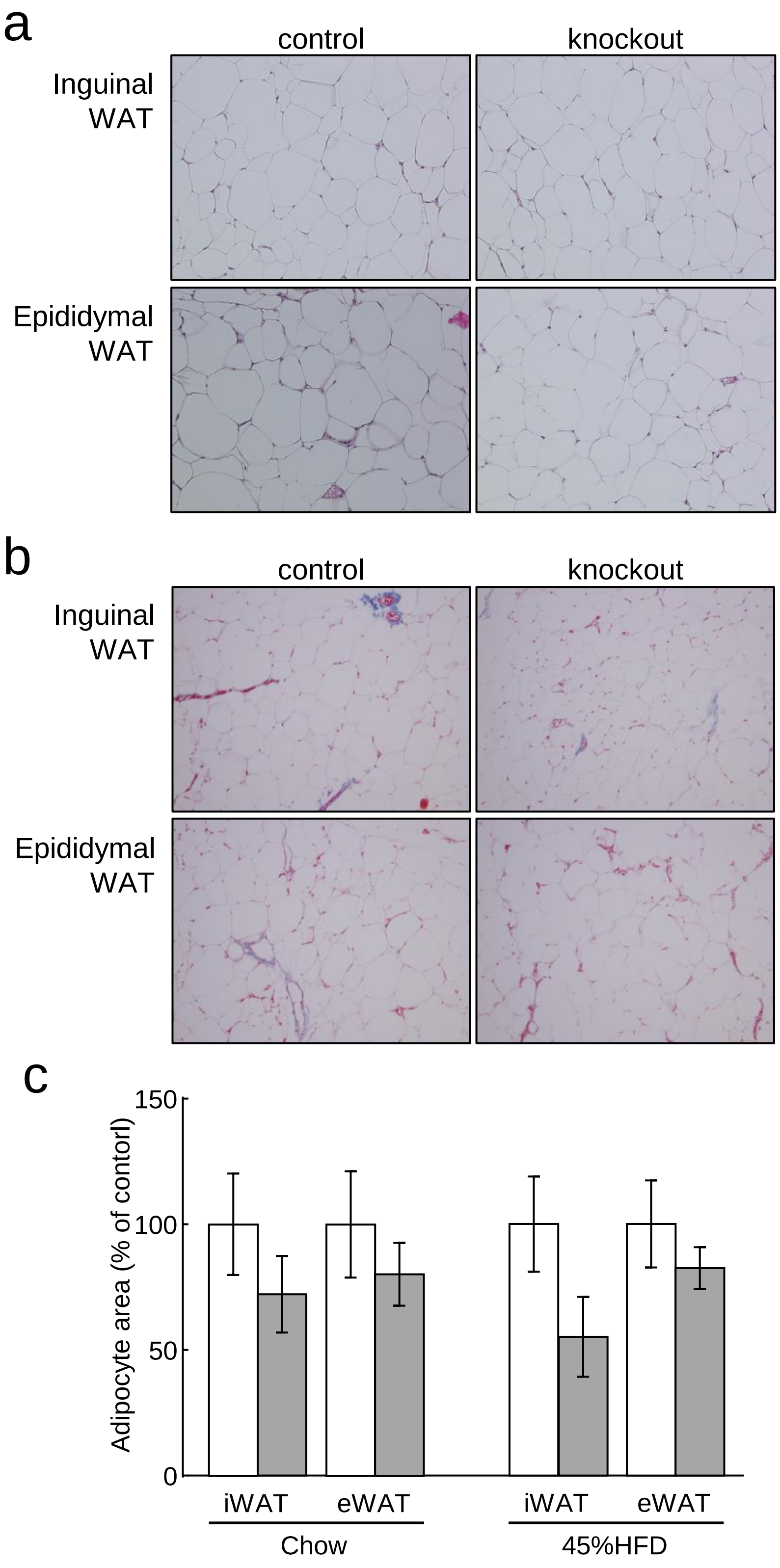

a
control
knockout
Inguinal WAT
Epididymal WAT
b
control
knockout
Inguinal WAT
Epididymal WAT
c
Adipocyte area (% of contorl)
150
100
50
0
iWAT
eWAT
Chow
iWAT
eWAT
45%HFD

ESM Fig. 2

ESM Fig. 2. Unchanged white adipose tissue morphology of *aP2-Bmpr1a*-KO mice. (**a**) Haematoxylin & eosin staining and (**b**) trichrome staining of iWAT and eWAT from control and *aP2-Bmpr1a*-KO mice. (**c**) Quantification of adipocyte area size from haematoxylin and eosin (H&E) staining of iWAT- and eWAT-sections derived from control and knockout mice maintained on a normal chow diet and an 45%HFD. White bars, control mice; grey bars, *aP2-Bmpr1a*-KO mice. Data are shown as means ± SEM.

ESM Fig. 3

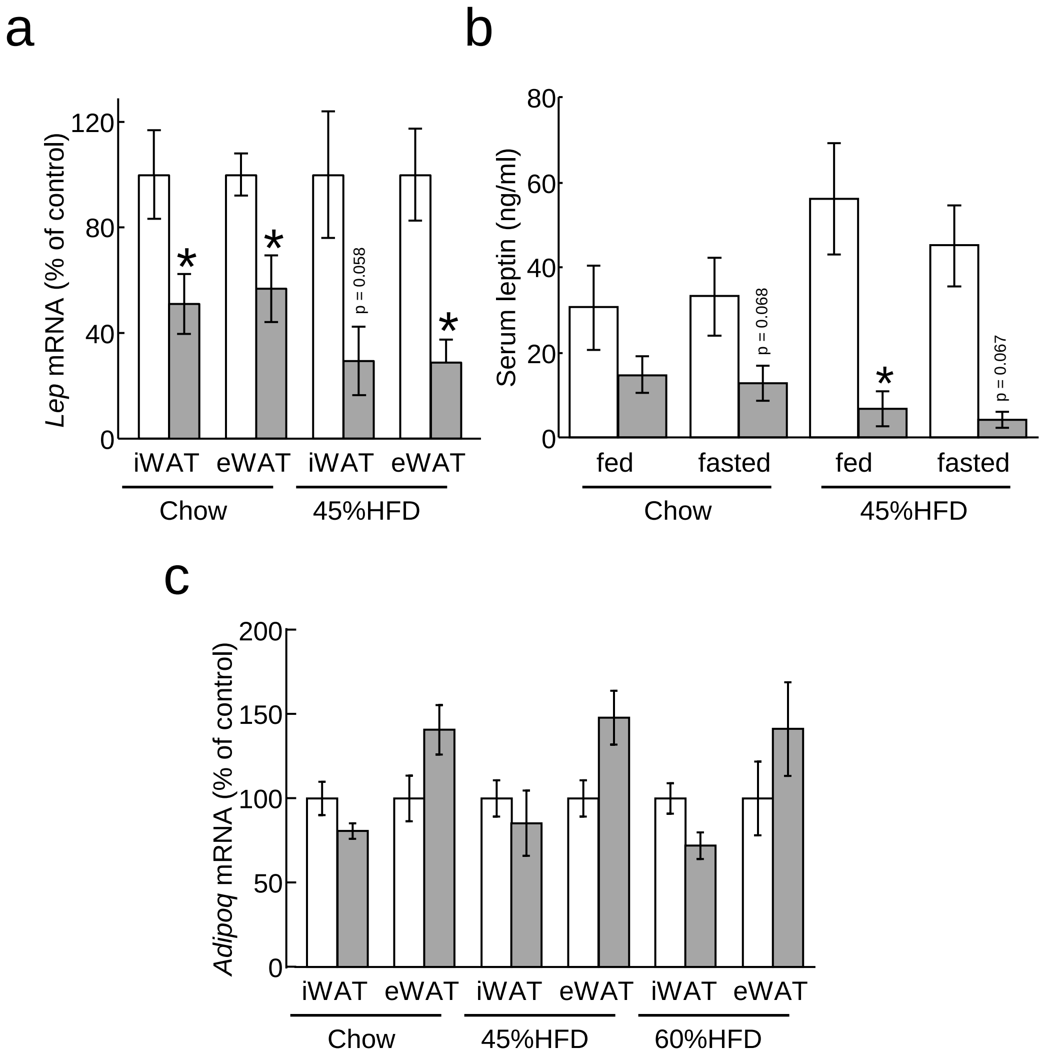
a
b
c
Lep mRNA (% of control)
120
80
40
0
*
*
p = 0.058
*
iWAT
eWAT
iWAT
eWAT
Chow
45%HFD
Serum leptin (ng/ml)
80
60
40
20
0
p = 0.068
*
p = 0.067
fed
fasted
fed
fasted
Chow
45%HFD
Adipoq mRNA (% of control)
200
150
100
50
0
iWAT
eWAT
iWAT
eWAT
iWAT
eWAT
Chow
45%HFD
60%HFD

ESM Fig. 3

ESM Fig. 3 Reduced BMP signalling in adipose tissue lowers circulating leptin level. (**a**) *Lep* mRNA levels in iWAT and eWAT in 52-weeks-old mice maintained on normal chow diet and on an 45%HFD. White bars, control mice; grey bars, *aP2-Bmpr1a*-KO mice. Data are shown as means ± SEM. (n=4-6 mice/ group; applies to all subsequent panels). (**b**) Serum leptin levels measured by ELISA in mice maintained on a chow diet or an 45%HFD with ad libitum food access (fed) or after a 16 hour overnight fast (fasted). (**c**) *Adipoq* mRNA levels in iWAT and eWAT in 52-weeks-old mice maintained on a normal chow diet, an 45%HFD and an 60%HFD. *$p<0.05$ compared with control mice.

ESM Fig. 4

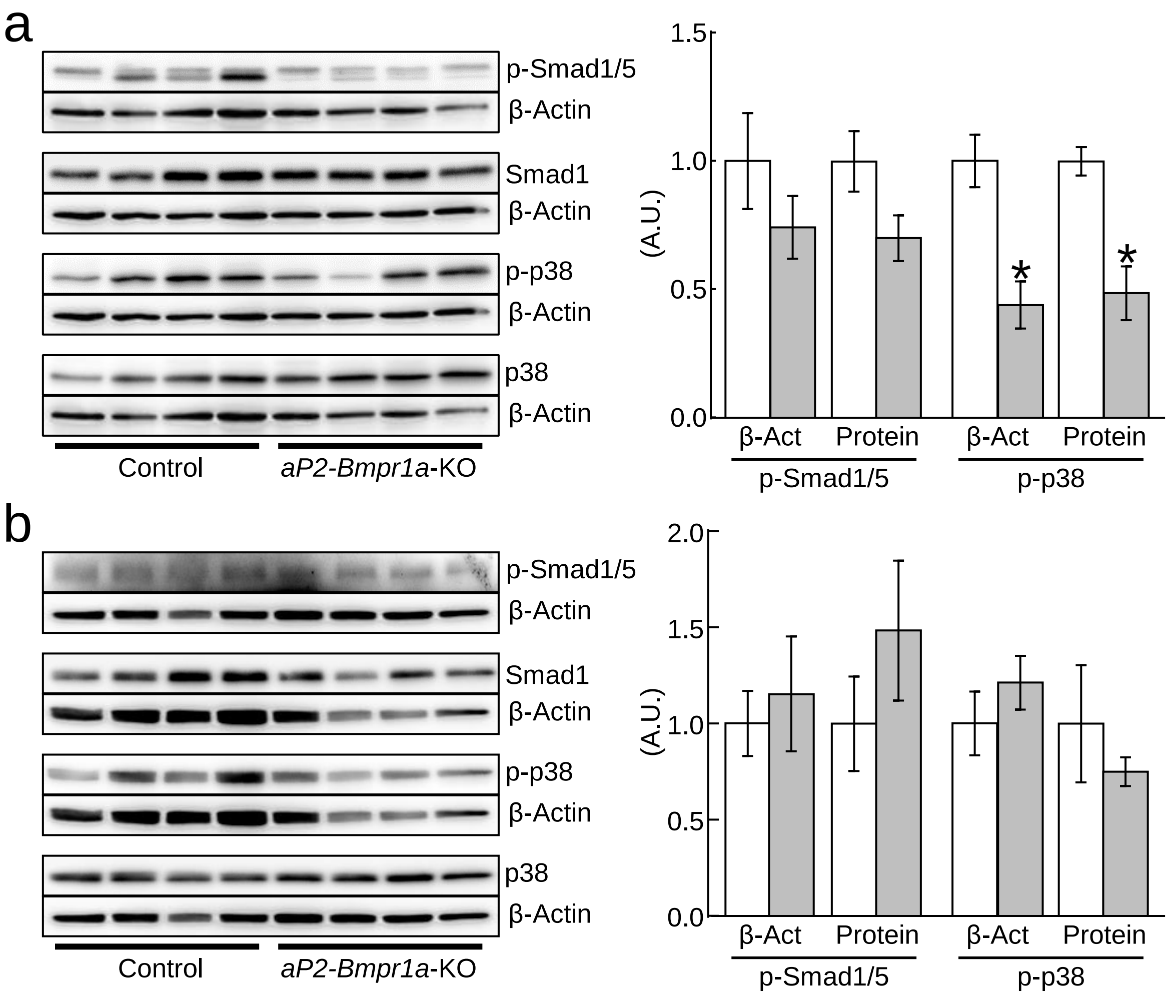
a
p-Smad1/5
β-Actin
Smad1
β-Actin
p-p38
β-Actin
p38
β-Actin
Control
aP2-Bmpr1a-KO
(A.U.)
1.5
1.0
0.5
0.0
β-Act
Protein
β-Act
Protein
p-Smad1/5
p-p38
*
*
b
p-Smad1/5
β-Actin
Smad1
β-Actin
p-p38
β-Actin
p38
β-Actin
Control
aP2-Bmpr1a-KO
(A.U.)
2.0
1.5
1.0
0.5
0.0
β-Act
Protein
β-Act
Protein
p-Smad1/5
p-p38

ESM Fig. 4

ESM Fig. 4 Analysis of basal activation levels of canonical BMP signalling pathways in white adipose tissues of *aP2-Bmpr1a*-KO mice. Western blot analysis of levels of phosphorylated Smad-1/5 (upper row: pSmad-1/5), basal Smad1 expression (second row: Smad1), phosphorylated p38-mitogen active protein kinase (third row: p-p38), and basal p38 expression (fourth row: p38) in eWAT (**a**) and iWAT (**b**) of mice maintained on normal chow until 52 weeks of age. White bars, control mice; grey bars, *aP2-Bmpr1a*-KO mice. For antibody-probing, the membrane was first probed against phosphorylated proteins, then stripped and re-probed with antibody directed against basal protein levels. To ensure no overlap with residual signal from previous antibodies, membranes that were initially used for detection of phosphorylated Smad1/5 were then probed with anti-p38 (basal), and *vice versa*. After stripping the membrane a second time, it was re-probed with antibody directed against β-actin. All protein levels were first normalised to β-Actin (β-Act) or total protein loaded per lane (‘protein’; image caption of membrane after Ponceau S staining after blotting transfer and before blocking step) as indicated. Ratios of phosphorylated protein to basal protein were then calculated (shown in quantifications on right). *$p<0.05$ compared with control mice of the same tissue.

ESM Fig. 5

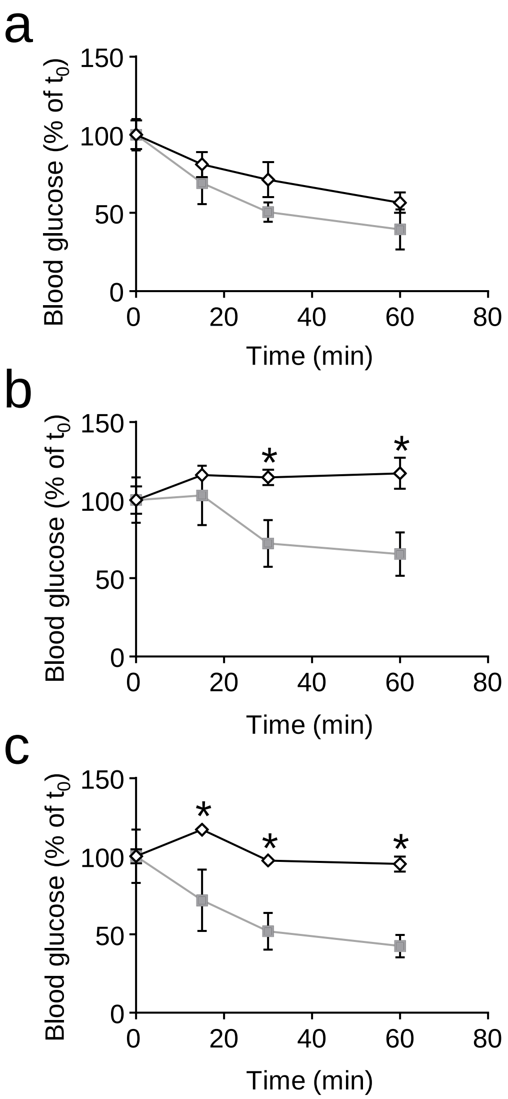
a
150
100
50
0
Blood glucose (% of t0)
0
20
40
60
80
Time (min)
b
*
*
150
100
50
0
Blood glucose (% of t0)
0
20
40
60
80
Time (min)
c
*
*
*
150
100
50
0
Blood glucose (% of t0)
0
20
40
60
80
Time (min)

ESM Fig. 5

ESM Fig. 5 Insulin tolerance testing in *aP2-Bmpr1a*-KO mice maintained on an 60%HFD throughout life. ITTs were conducted at (**a**) 22 weeks of age (18 weeks of 60%HFD) with $p=0.0952$ for AUC comparing the two genotypes, (**b**) 43 weeks of age (39 weeks on 60%HFD) with $p=0.0159$ for AUC comparing the two genotypes, and (**c**) 54 weeks of age (50 weeks on an 60%HFD) with $p=0.0159$ comparing AUC for the two genotypes. Open diamonds, controls; grey squares, *aP2-Bmpr1a*-KO. Data are shown as means ± SEM ($n=4$ for control, $n=5$ for *aP2-Bmpr1a*-KO). *$p<0.05$ compared with control mice at the given time point.

ESM Fig. 6

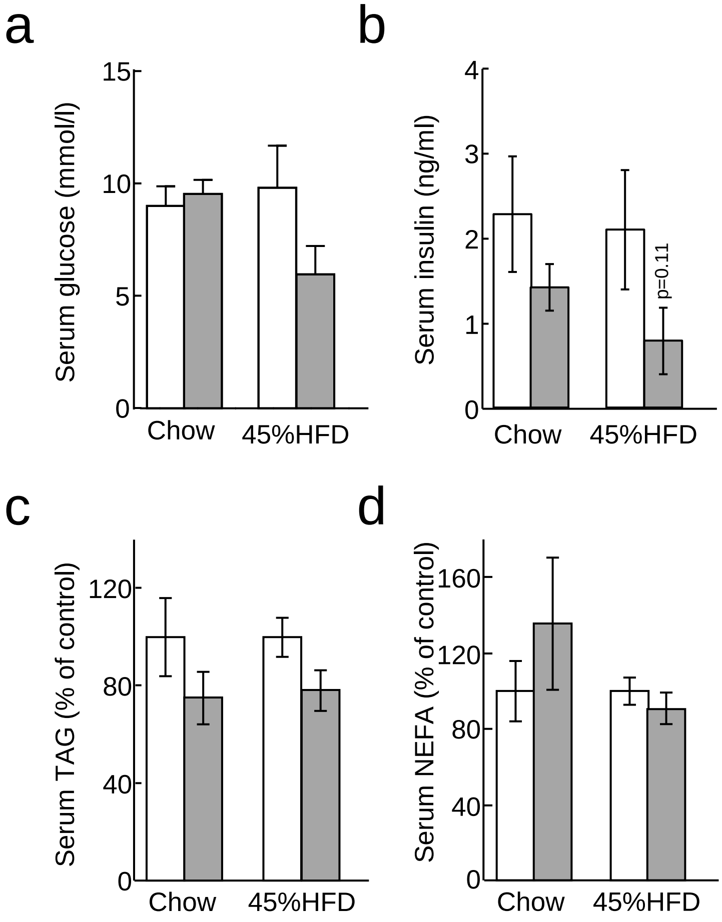

ESM Fig. 6 Serum parameters of *aP2-Bmpr1a*-KO mice. (**a**) Serum glucose levels, (**b**) serum insulin levels, (**c**) serum triacylglycerol (TAG) levels, and (**d**) serum NEFA levels in 52-weeks-old mice with ad libitum access to normal chow (Chow; n=6/genotype) or an 45%HFD (n=4 for control mice, n=6 for *aP2-Bmpr1a*-KO mice). White bars, control mice; grey bars, *aP2-Bmpr1a*-KO mice. Data are shown as means ± SEM. *p<0.05 and **p<0.01 compared with control mice.

ESM Fig. 7

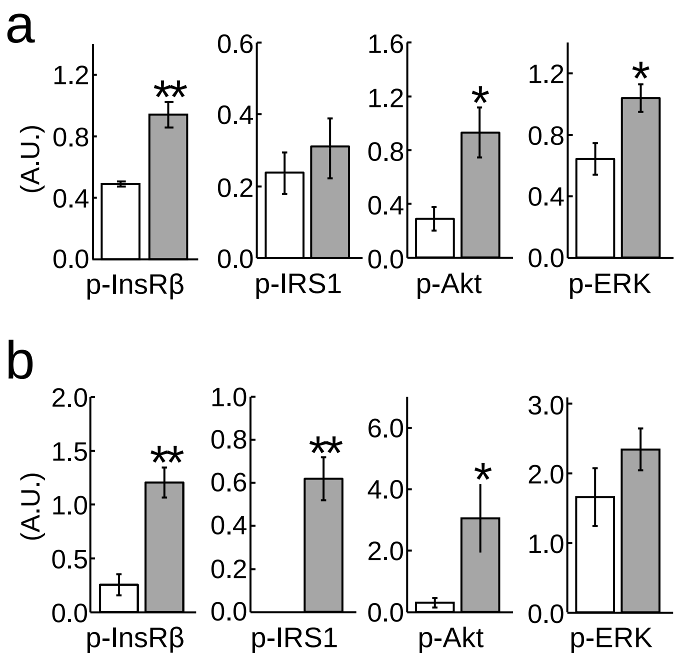

ESM Fig. 7 Western blot quantification of insulin signalling components in white adipose tissues after insulin stimulation (for Figure 2). Western blot analysis of insulin-stimulated activation of the insulin signalling cascade in iWAT(**a**) and eWAT (**b**) of control and *aP2-Bmpr1a*-KO mice that were maintained on an 60%HFD prior to injection of insulin. White bars; grey bars, *aP2-Bmpr1a*-KO mice. Levels of the phosphorylated forms of insulin receptor-β (p-InsRβ), insulin receptor substrate (p-IRS-)1, protein kinase B (p-AKT), and extracellular-signal regulated kinase (p-ERK) were detected and normalised to basal expression of β-tubulin (β-Tub). Images were captured using conventional luminescence-based imaging techniques and analysed using ImageJ software. Data are shown as means ± SEM. *p<0.05, **p<0.01 and ***p<0.001 compared to control mice of the same treatment group and/or tissue type.

ESM Fig. 8

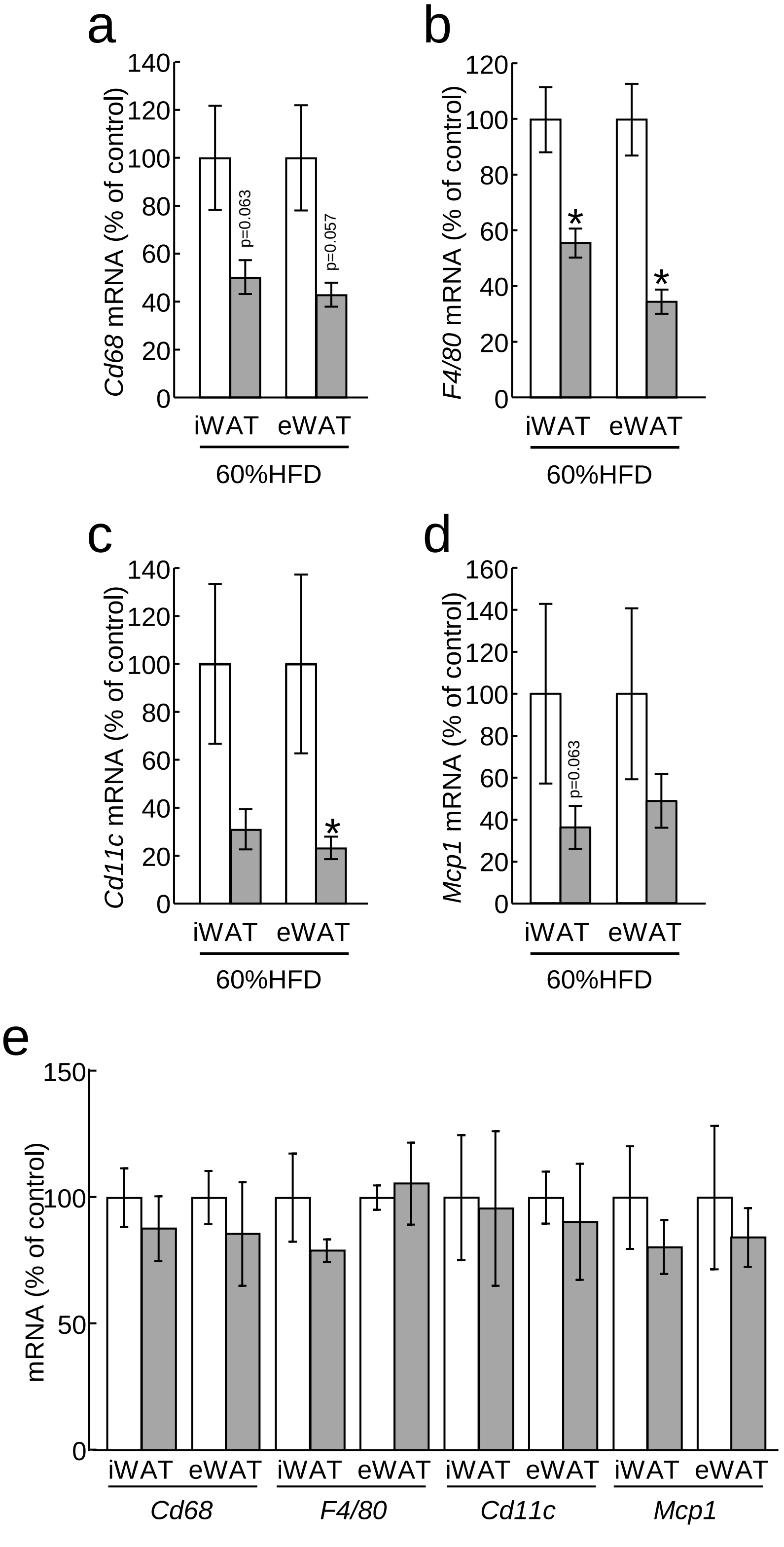
a
b
c
d
e
Cd68 mRNA (% of control)
F4/80 mRNA (% of control)
Cd11c mRNA (% of control)
Mcp1 mRNA (% of control)
mRNA (% of control)
p=0.063
p=0.057
*
iWAT
eWAT
60%HFD
Cd68
F4/80
Cd11c
Mcp1

ESM Fig. 8 Expression of immune cell and pro-inflammatory markers. Gene expression analysis of macrophage markers *Cd68* (**a**), *F4/80* (**b**), *Cd11c* (**c**), and *Mcp1* (**d**) in iWAT and eWAT of mice maintained on an 60%HFD until 54 weeks of age. White bars, control mice; grey bars, *aP2-Bmpr1a*-KO mice. (**e**) Gene expression of the same genes in young, 12-weeks-old mice maintained on a normal chow diet. Data are shown as means ± SEM (n=4-5/group). $^{*}p<0.05$ compared to control mice of the same treatment group and/or tissue type.

ESM Fig. 9

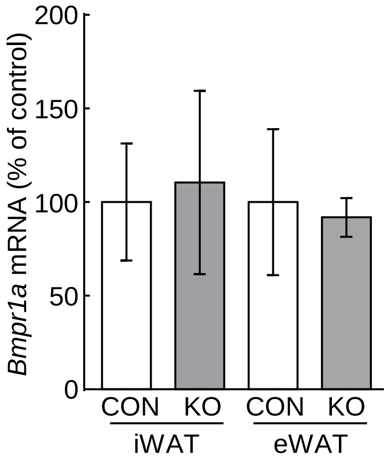

ESM Fig. 9 *Bmpr1a* gene expression in sorted macrophages in *aP2-Bmpr1a*-KO mice. *Bmpr1a*-mRNA levels in FACS-purified macrophages (surface markers: CD45+;CD11b+; F4/80+;CD3e-;CD19-;CD49b-;Ter119-) isolated from iWAT and eWAT of mice maintained on an 60%HFD. White bars, control; grey bars, *aP2-Bmpr1a*-KO. Data are shown as means ± SEM.

ESM Fig. 10

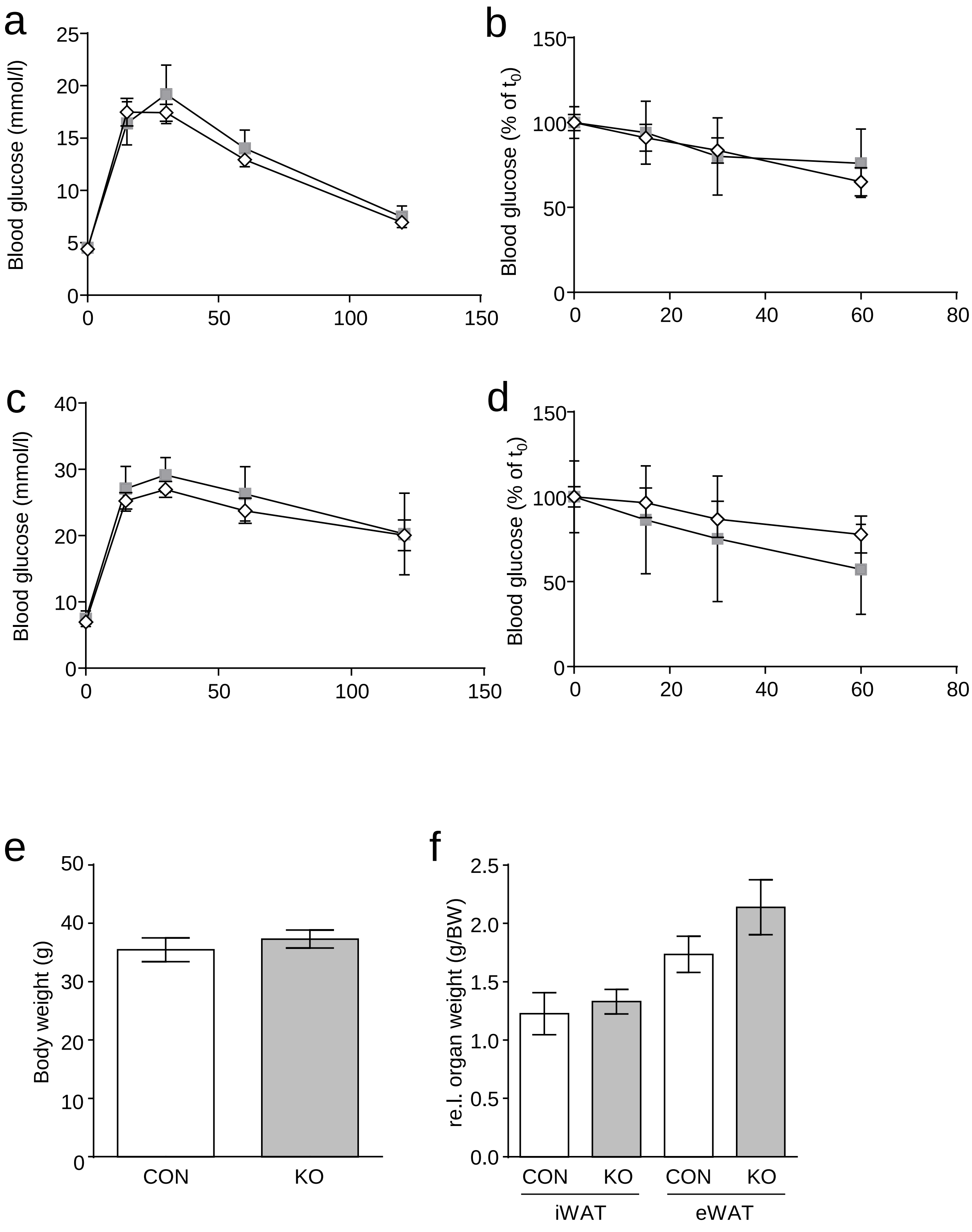
a
Blood glucose (mmol/l)
25
20
15
10
5
0
0
50
100
150
b
Blood glucose (% of t_0)
150
100
50
0
0
20
40
60
80
c
Blood glucose (mmol/l)
40
30
20
10
0
0
50
100
150
d
Blood glucose (% of t_0)
150
100
50
0
0
20
40
60
80
e
Body weight (g)
50
40
30
20
10
0
CON
KO
f
re.l. organ weight (g/BW)
2.5
2.0
1.5
1.0
0.5
0.0
CON
KO
CON
KO
iWAT
eWAT

ESM Fig. 10

ESM Fig. 10 Phenotyping of *Adipoq-Bmpr1a*-KO mice. Glucose tolerance (**a**) and insulin tolerance testing (**b**) in *Adipoq-Bmpr1a*-KO mice maintained on a normal chow diet. Open diamonds, control; grey squares *Adipoq-Bmpr1a*-KO mice (n=9 for control and n=7 for knockout). Also applies to subsequent panels. Glucose tolerance (**c**) and insulin tolerance testing (**d**) in mice maintained on an 60%HFD (n=7/group). (**e**) Body weight and (**f**) WAT pad weights in *Adipoq-Bmpr1a*-KO mice on normal chow diet at approximately 8 months of age. White bars, control; grey bars, knockout mice (n=9 for control mice, n=7 for *Adipoq-Bmpr1a*-KO mice). Data are shown as means ± SEM.

ESM Fig. 11

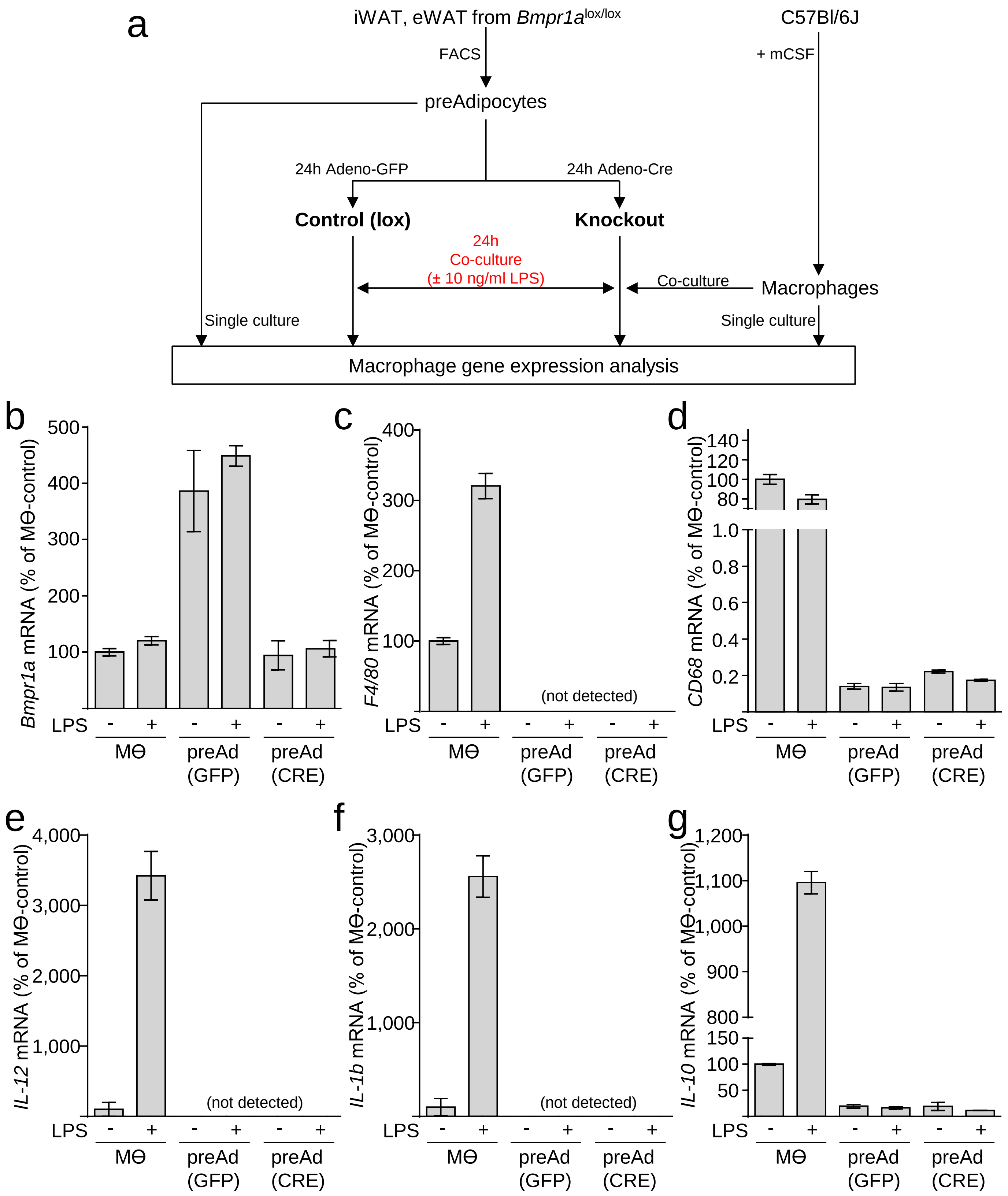
a
iWAT, eWAT from Bmpr1a lox/lox
FACS
C57Bl/6J
+ mCSF
preAdipocytes
24h Adeno-GFP
24h Adeno-Cre
Control (lox)
Knockout
24h
Co-culture
(± 10 ng/ml LPS)
Co-culture
Macrophages
Single culture
Single culture
Macrophage gene expression analysis
b
Bmpr1a mRNA (% of MΘ-control)
c
F4/80 mRNA (% of MΘ-control)
(not detected)
d
CD68 mRNA (% of MΘ-control)
e
IL-12 mRNA (% of MΘ-control)
(not detected)
f
IL-1b mRNA (% of MΘ-control)
(not detected)
g
IL-10 mRNA (% of MΘ-control)
LPS - + - + - +
MΘ
preAd (GFP)
preAd (CRE)

ESM Fig. 11

ESM Fig. 11 Co-cultivation of macrophages with *Bmpr1a*-KO pre-adipocytes reduces macrophage activation. (**a**) Schematic overview of co-culture experimental setup. Pre-adipocytes and macrophages were isolated separately. Macrophages were treated with 50 ng/ml of macrophage colony-stimulating factor (mCSF) throughout culture. Pre-adipocytes were infected with respective adenoviruses for 24 hours. Adenovirus was washed off the plates and cells were allowed to expand for several days in the absence of viral particles. Gene expression analysis of *Bmpr1a* (**b**), *F4/80* (**c**), *Cd68* (**d**), *Il12* (**e**), *Il1b* (**f**), *Il10* (**g**) in single cultures of macrophages (MΘ), pre-adipocytes derived from eWAT that were either infected with a control adenovirus expressing green fluorescent protein (GFP), or with adenovirus expressing Cre-recombinase (CRE). Gene expression was measured in untreated cells (-) or in cells treated with 10 ng/ml of lipopolysaccharide (LPS; +) prior to harvest. Data are shown as means ± SEM.
